# Fused Filament Fabrication Based on Polyhydroxy Ether (Phenoxy) Polymers and Related Properties

**DOI:** 10.3390/polym13101549

**Published:** 2021-05-12

**Authors:** Christian Brauner, Marco Küng, Delal Arslan, Christoph Maurer

**Affiliations:** Institute of Polymer Engineering, FHNW University of Applied Sciences and Arts Northwestern Switzerland, Klosterzelgstrasse 2, 5210 Windisch, Switzerland; marco.kueng@fhnw.ch (M.K.); delal.arslan@students.fhnw.ch (D.A.); christoph.maurer@fhnw.ch (C.M.)

**Keywords:** fused filament fabrication, fused deposition modeling, phenoxy, composite, 3D printing, additive manufacturing

## Abstract

This paper describes the first-time application of polyhydroxy ether polymers, so-called phenoxy, to fused filament fabrication (FFF). Phenoxy is an amorphous thermoplastic polymer that is based on the same building blocks as epoxide resins. This similarity creates some unique properties such as dissolution to epoxide systems, which is why phenoxy is used as an additive for toughening. In this study, the processing parameters were characterized, a filament was extruded and applied to FFF printing, and the final mechanical characteristics were determined. The study concludes with a comparison with other standard FFF materials.

## 1. Introduction

Additive manufacturing (AM) is a type of manufacturing that is becoming increasingly accepted in industry. An average growth of 25% per year is expected in the AM market. Previously, this type of production was used more for prototypes, but more and more companies are planning to adopt this technology in their series production [1]. A forecast shows that 46% of global companies even want to produce end products with AM. Today, many industries, such as aviation, automotive, and consumer goods, are already working with AM as standard—and the trend is increasing [1]. It is noteworthy that in the aviation and consumer goods industries, all companies surveyed want to integrate AM into their production. Seventy percent of the companies surveyed using this technology worked with polymers in 2019, and 50% worked with metal [1]. In addition, this technology enables series production, in which individual adaptation is possible for different customers [1].

The fused filament fabrication (FFF) method is one of the most used additive manufacturing methods for polymer materials based on direct material melting beside vat photopolymerization (SLA, DLP, CDLP), sheet lamination (LOM), direct energy deposition (EBAM), binder jetting (BJ), material or polymer jetting technologies [2] or powder bed fusion (Selected Selective Laser Sintering, EDM, MJF) [3]. In the area of the individual material, a thermoplastic polymer is brought to a flowable state, pressed through a nozzle, and deposited on a previously defined location. As a result, there is almost no scrap material, which makes the process efficient.

The disadvantage of FFF is that the component is provided with a weak point. There are no identical bonds between the layers that are deposited as within these layers [4,5]. The result is severe anisotropy. This makes the structure susceptible to shear forces, as well as to a load orthogonal to the printing level. Therefore, the component strengths depend on the orientation of the deposited layers, temperature profile, feed forces, and further processing parameters [5]. Given the low strengths of individual materials, the process is only used in industry in the production of prototypes. However, due to its simplicity and diversity, many modern applications are encountered in the hobby space [6].

This study describes the first-time application of polyhydroxy ether polymer, so-called phenoxy, in fused filament fabrication. In a literature review, it was found that these polymers have been used in the past by melt spinning [7], extrusion, or as an additive in thermoset resins to improve fracture toughness [8]. Phenoxy is an amorphous thermoplastic polymer that is based on the same building blocks as epoxide resins (bisphenol A and epichlorohydrin). Phenoxy was developed in the early 1960s by Union Carbide (UCAR), but it was not commercialized at that time. In the 1980s, the company name changed to UCAR phenoxy resin, and the first development of phenoxy dispersion was commercialized. In 1993, the business was acquired by Phenoxy Associates, and the polymer name changed to PAPHEN phenoxy resin. In 1997, the business was acquired by InChem and changed to INCHEMREZ phenoxy resin; in 2016, the Phenoxy business line was sold to Gabriel Performance Products and renamed to Gabriel Phenoxies Inc., and recently, in 2021, GabrielChem was acquired by Huntsman Advanced Materials.

**Figure 1:**
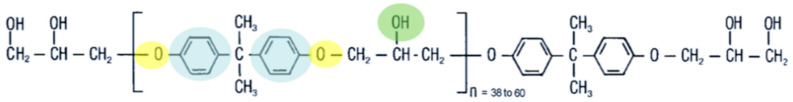
Polymer structure of phenoxy.

Phenoxy is synthesized from bisphenol A and epichlorohydrin. The structure shown in Figure 1 shows the amorphous thermoplastic properties such as rigidity, thermal and chemical stability, and adhesion strength. The rigidity and thermal stability result from the aromatic compounds (blue), the chemical stability of the oxygen atoms in the main chain (yellow), and the adhesion strength of the hydroxyl groups (green). Phenoxy resins can also be crosslinked with phenolic resins by reacting to their functional hydroxyl group. In doing so, they develop a chemical resistance and a certain hardness. They also adhere to steel, aluminum, glass fibers, carbon fibers, nylon, and polyester [7].

Phenoxy polymers are commercially available in different forms such as emulsion, powders, films, and granulates.

In this study, a grade for extrusion was selected as most promising candidate for FFF processing. This study reports the determination of the characterization of the processing parameters, filament extrusion, application in FFF printing, and determination of final mechanical tensile properties. The study concludes with a comparison to other standard FFF materials.

## 2. Material Characterization

For the investigation in this study, the Phenoxy grade PKHB+ XLV (Huntsman Advanced Materials) was selected. The material PKHB+ XLV had a molecular weight of 37,000 g/mol [7]. Phenoxy PKHB+ XLV has a low viscosity and percent solids when compared to most available phenoxy resins such as PKHH (52,000 g/mol) and PKFE (ultra-high molecular weight 60,000 g/mol).

In the following section, different parameters such as the glass transition, thermal degradation, and viscosity are outlined, which are relevant for the processing of filaments as well for the FFF process.

### 2.1. Analysis of Glass Transition Temperature with Differential Scanning Calorimetry (DSC)

**Figure 2:**
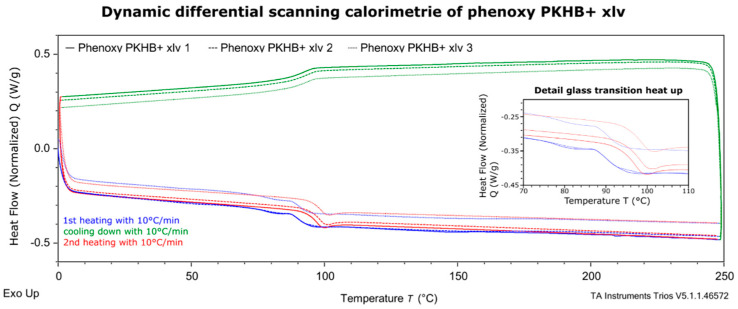
Dynamic differential scanning calorimetry (DSC) curve for the determination of the glass transition temperature. Blue: first heating, red: second heating and green cooling.

In a first step, the material was analyzed via differential scanning calorimetry (DSC) using a DSC 25 from TA instruments. Three samples of around 8 mg were heated at a constant heat rate of 10 °C/min from 0 to 250 °C (blue). This was repeated (red line) with the same sample. Figure 2 shows the derived heat flow over the temperature range. For the measurement, granules were used.

In the related characterization test, the glass transition temperatures changed initially from 90.67 ± 0.17 °C in the first run to 96.65 ± 0.93 °C in the second run. The curves of the first heating differ in the temperature of the glass transition temperature and in the shape of the glass transition temperature. The difference in shape, the small endothermic peak, is due to enthalpy relaxation, which may be caused by rapid cooling during initial manufacturing of the granulates.

### 2.2. Thermogravimetric Analysis (TGA)

In the second step, the material was subjected to thermogravimetric analysis (TGA) using a TGA Q500 from TA instruments. Three samples with weights between 10 and 15 mg were heated with a constant heat rate of 10 °C/min from 20 to 700 °C in nitrogen atmosphere and from 700 to 1000 °C in air to analyze thermal degradation.

**Figure 3:**
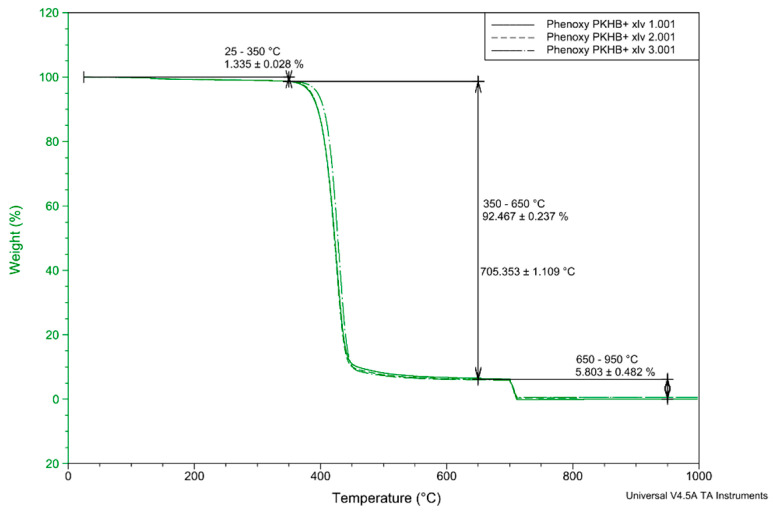
Thermogravimetric analysis (TGA) characterization for degradation.

As can be seen from Figure 3, the sample loses 1.335 ± 0.028% of its mass between 25 and 350 °C due to degassing and/or moisture loss. Between 350 and 650 °C, the sample loses another 92.467 ± 0.237% of mass. Here, the structure of the sample decomposes, and individual components degas. It is left with 5.803 ± 0.482% of carbon, which then burns under the air atmosphere. In total, a share of about 99.605% of the mass escapes. The remaining mass could be soot particles formed during combustion and still adhere to the crucible. Since only one stage can be seen in the result, it can be assumed that there are no further additives in the granules.

### 2.3. Analysis of the Temperature-Dependent Flow Behavior through Rheometric Studies

In rheology, one considers the flow behavior of the polymer melt. On the one hand, polymer melts have a structurally viscous behavior. This means that the viscosity decreases with increasing shear rate. Due to the increasing shear of the material, more entanglements are loosened, allowing the molecular chains to move more freely. This leads to decreased resistance of the polymer melt and, with it, the viscosity. In the FFF process, the apparent shear rate on the wall ($\dot{\gamma })$ depends on the nozzle diameter and the printing speed [9]. This can be expressed by the rheological Equation (1), where the volume flow $\left(\dot{V}\right)$ can be expressed by the velocity in the nozzle (v), which corresponds to the printing speed and the area of the nozzle diameter (A). The formula can be further simplified by expressing the nozzle area by the nozzle radius (R).

(1) $$\dot{\gamma }=\frac{4\cdot \dot{V}}{\pi \cdot R^{3}}=\frac{4\cdot v\cdot A}{\pi \cdot R^{3}}=\frac{4\cdot v}{R}$$

**Figure 4:**
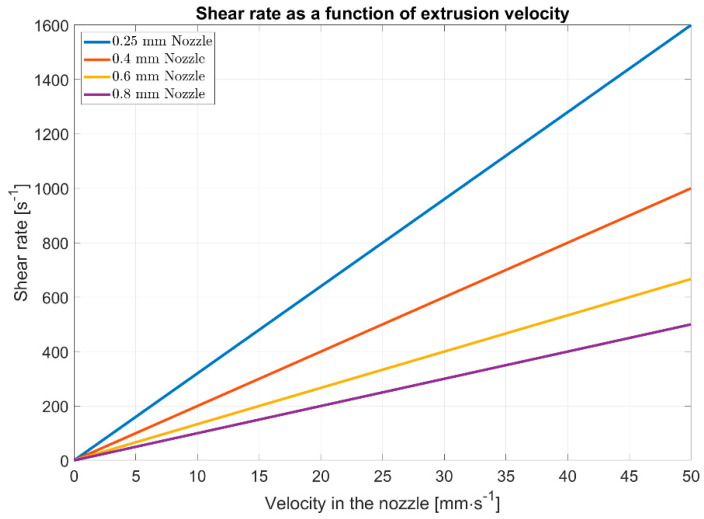
Shear rates in fused filament fabrication (FFF) related to different nozzle diameters.

Due to the acceleration at the beginning of the print path or after curves, a change in the speed of the printhead occurs, resulting in a varying volume flow and thus a varying speed in the material feed. Figure 4 shows the shear rates that occur depending on the standard FFF nozzle diameters and the feed rates.

The viscosity, depending on the temperature and the shear rate, was determined using a plate-to-plate setup with an MCR 300 rheometer from Physica. A plate diameter of 25 mm with a gap of 1 mm and temperatures of 190, 200, and 210 °C were applied. To determine the shear rate, an excitation of 0.1 to 100 rad/s was applied.

**Figure 5:**
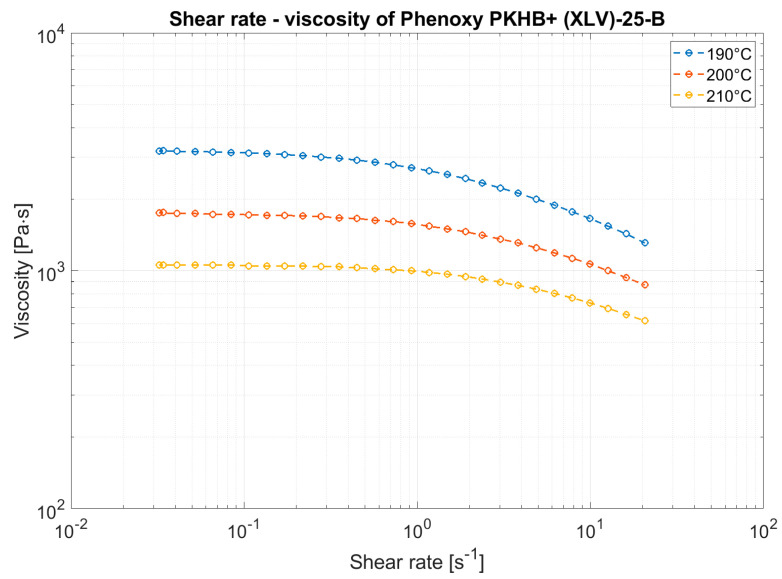
Viscosity behavior measured with a plate-to-plate rheometer.

The results (Figure 5) show that the viscosity decreases from a critical shear rate of 1 s^−1^. Thus, so-called shear thinning occurs, which can be assigned to the structurally viscous behavior. It can also be observed that as the temperature rises, the viscosity also decreases. This is because as the temperature rises, the molecular chain freedom of movement increases, and it can thus more easily detach itself from entrenchments and hooks. In the area of the Newtonian plateau, the shear forces are too low to release the entrenchments. Therefore, the viscosity in this area does not decrease with increased shear rate. The viscosity within this range is called zero viscosity. As shown in Figure 5, far higher shear rates occur in the FFF process and in the extrusion process. Therefore, rheological behavior at higher shear rates would have to be investigated. Nevertheless, it can be obtained from this examination that shear dilution is to be expected during the FFF process or extrusion.

## 3. Filament Production

**Figure 6:**
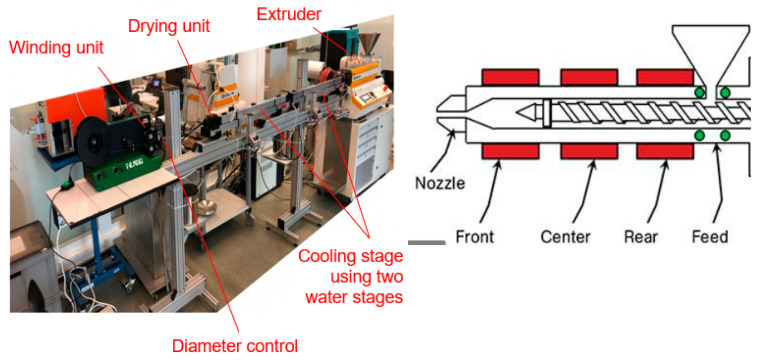
Filament extrusion line at Institute of Polymer Engineering, FHNW University of Applied Sciences and Arts, Northwestern Switzerland.

The filament production was carried out on a self-assembled extrusion line using a Collin Teach line extruder, a cooling stage using two water reservoirs, a drying unit, a diameter control (Mitutoyo/Filalogger), and a winding unit from Filabot (FB00073); see Figure 6. The nozzle diameter was 2 mm, finally obtaining a filament diameter of 1.75 mm. In general, most of the FFF printers use filaments of 2.85 mm or 1.75 mm. In this study, a small filament was selected based on the availability of the extrusion dye.

Table 1 summarized the used parameters for the filament extrusion applying temperatures of 195 °C in the extruder [3], a extruder speed of 35 U/min, and a pressure of 42 bar.

**Figure 7:**
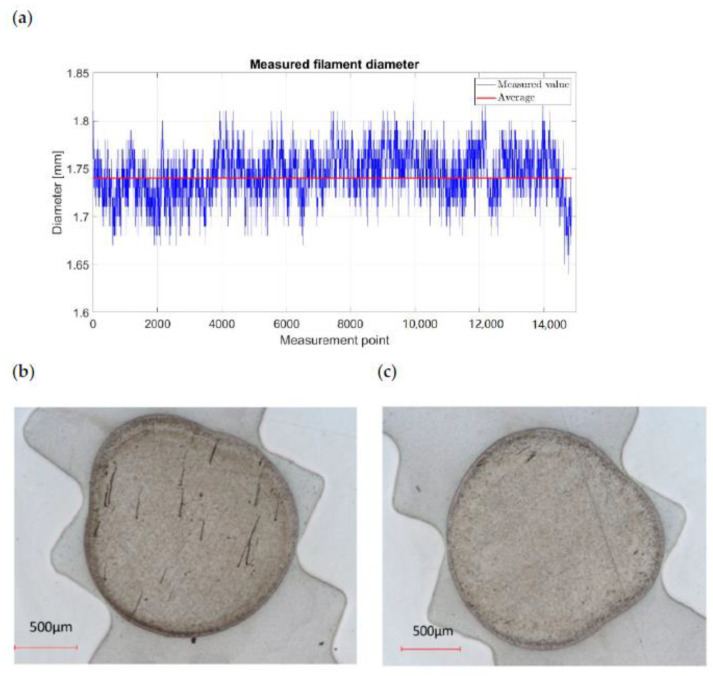
(**a**) Measurement of filament diameter, (**b**,**c**) shape of the produced filament.

The following diagram shows the control of the measured diameter, and the average is around 1.74 mm, with a standard deviation of ±0.09 mm. The shape of the filament was controlled via microsection analysis, and a small shape deviation was detected, but in the final FFF printing, this deviation did not create any problems (Figure 7). Out-of-roundness or a diameter that is too large can lead to an interruption in the FFF process due to clogging in the printhead. The filaments did not show any voids.

In general, it can be summarized that extrusion was quite stable with an output rate of 1.6 kg/h, a pulling speed of 0.15 m/s, and an applied shear rate of 300 s^−1^, and the targeted diameter was achieved.

## 4. Specimen Manufacturing and Testing

To evaluate the mechanical performance of the material, mainly stiffness and strength, and to evaluate the process dependency tensile test have been performed in XY and Z direction to the main print path.

**Figure 8:**
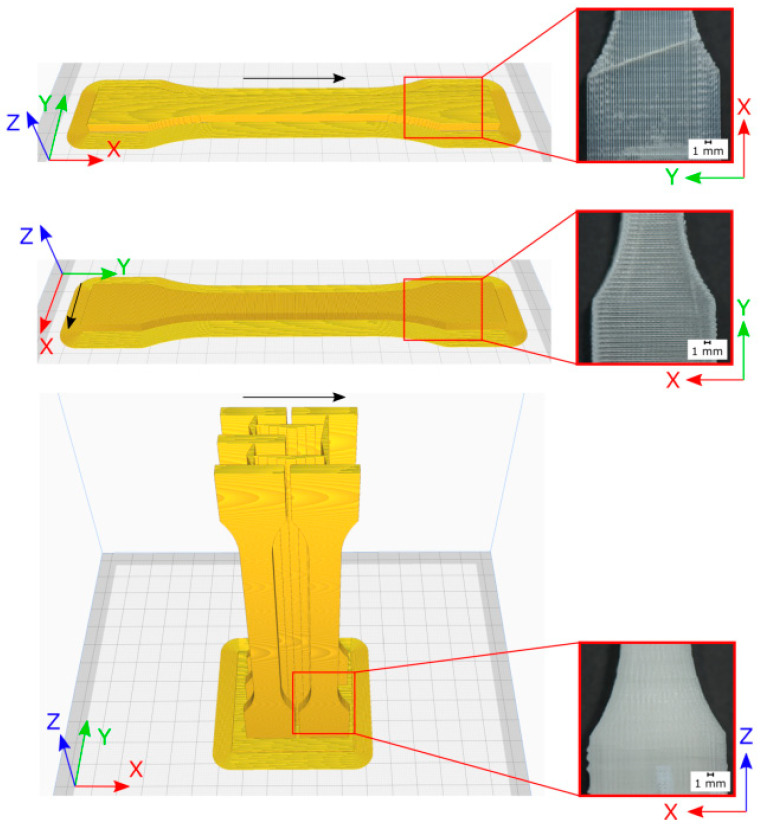
Printed tensile bars with coordinate system (**left**) and the related visual quality (**right**), black arrow shows printing direction.

The material was used to print classical tensile bars applying only unidirectional print paths in accordance with DIN EN ISO standard 527-1 (Figure 8). These tests have the advantage to apply uniaxial loading conditions in the direction of the material coordinate system (X, Y, and Z) in contrast to bending test. The main printing direction is defined as the X direction, Y is transverse in plane, and Z is the out of plane direction as visible in Figure 8. These test samples were analyzed by microsection analysis of cross-sections, tensile tests, and dynamic mechanical analysis to derive the temperature-dependent storage modulus. As a reference and baseline, the results were compared to an injection-molded specimen manufactured from the same material.

In FFF, a thermoplastic in the form of a filament is pressed from a conveying unit into a heated nozzle and made flowable, and then, the polymer melt is deposited at a previously defined location. This creates a three-dimensional geometry layer by layer. This process results in strong anisotropy in all properties. Many influences characterize the mechanical properties of the component. The influences can be divided into the following three categories:

- Structure parameters: infill, construction orientation, grid orientation, layer height, layer width, air gap, and part geometry;
- Manufacturing parameters: temperature gradient, nozzle temperature, printing speed, nozzle diameter, and humidity;
- Material: material properties.

There are three different types of construction orientation. The tensile strengths of the component differ greatly due to different binding forces. The decisive factor for the binding force is the diffusion between the discarded strands. Diffusion between adjacent strands within the same layer leads to better results. Since these strands were deposited one after the other, the temperature is higher along with the diffusion between them. In the case of adjacent strands from different layers, the diffusion is therefore worse, since these are not deposited one after the other, and thus, the already cooled strand leads to lower diffusion [2,10]. Therefore, it is important whether the bond between the strands of different layers, the connection between the strands of the same layer, or the strand itself is claimed. Therefore, the tensile strength is lowest when a component is applied orthogonally to the printing level. The bindings between the strands of different layers are called interlayer bindings. The bonds between the strands of the same layer are called trans-layer bindings. The tensile test specimens, which are oriented in angular and flat construction, show the highest tensile and bending stiffnesses [2,9].

This approach was applied as the first assessment of properties with the following printing parameters. An Ultimaker 2+ Extended from Ultimaker was used with an E3D Titan Aero 1.75 12v and a 1.75 mm E3D v6 Nozzle X. Three different unidirectional layer setups were used to determine the process-induced mechanical properties. This allows the determination of the mechanical properties orthogonal to the printing direction and to the printing direction. Table 2 summarizes the used printing parameters. The layer structure is shown in Figure 8.

**Figure 9:**
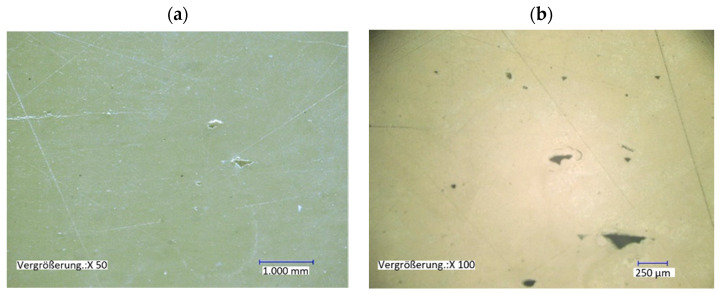
Micrographs of 3D-printed tensile test bars in the Y-direction, (**a**) wall section of the tensile test bar, (**b**) Detail view of the porosity found.

The following Figure 9 shows a microsection analysis of the printed specimens. Almost no porosity is visible. The process-induced print paths are not apparent.

**Figure 10:**
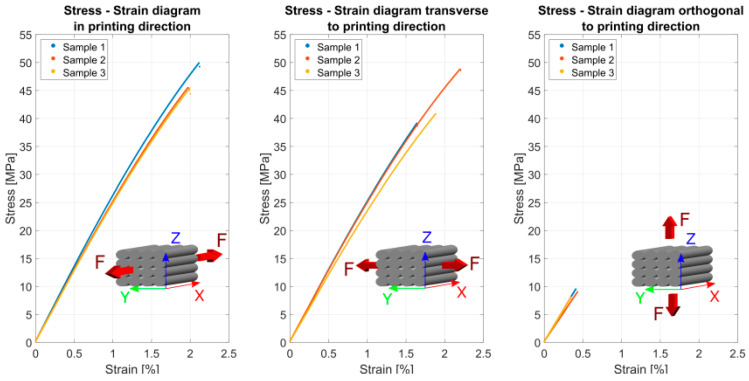
Tensile test results according to DIN EN ISO standard 527-1 of printed samples.

Tensile testing according to DIN EN ISO standard 527-1 was performed on a Zwick 100 kN Allround floor-standing, and the following Figure 10 shows the stress–strain relationship, the tensile strength, fracture strain, and Young’s modulus. According to the standard, a minimum of three samples with a test speed of 2 mm/min has to be tested.

Table 3 summarizes the used the achieved mechanical stiffness, fracture stress, and fracture strain related to the printing direction.

The printing direction has a high influence on the resulting properties, but the tensile strength in the fiber direction is quite high, with an average of 48 ± 3.17 MPa, compared to the transverse directio2n, with an average of 41 ± 6.0 MPa, and the orthogonal direction, with an average of 9 ± 0.74 MPa. This means a reduction in the tensile strength by the factor of 5.3, respectively 4.5. If this result is compared to similar study using PLA, a factor of 2.6 to 4 can be found [3]. The Young’s modulus is less influenced by the printing direction, with average values of 2590 ± 77.3 MPa in the printing direction. Tests in the direction transverse to the print path result in an average stiffness of 2500 ± 87.9 MPa, with a difference of 3.5%. In the out-of-plane direction, the average Young’s modulus is reduced by 15%, with average results of 2200 ± 137 MPa.

To interpret this experimental performance, a comparison was performed with injection-molded tensile bars. The reason for this was based on the missing reference data in the literature and datasheet for this polymer grade phenoxy PKHB+ XLV.

Tensile bars were manufactured by injection molding and tested as well, and the results are shown in the next figure. The tensile tests were carried out according to DIN EN ISO 527-1 standard, and five tensile test bars were tested.

**Figure 11:**
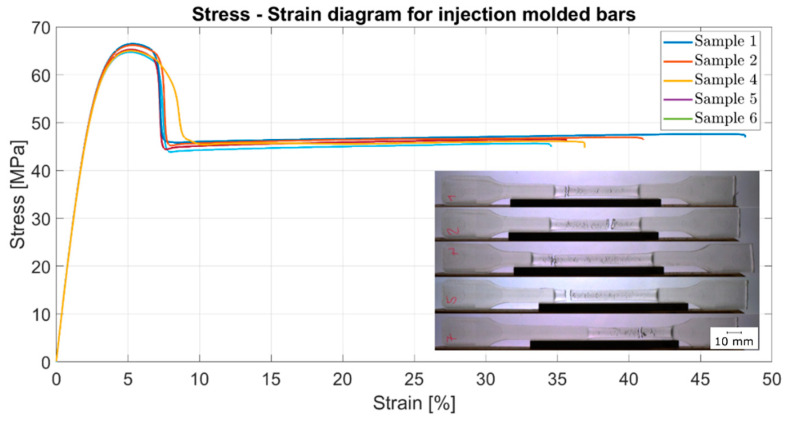
Tensile test results according to DIN EN ISO standard 527-1 of injection-molded samples.

The stress–strain curve (see Figure 11) shows the behavior of a ductile material with a yield point in contrast to the same material process by FFF. This is also evident from the tested specimens, due to the stretching between the clamping points. As visible, the properties of injection-molded tensile bars are higher with a tensile strength of 65.5 ± 0.76 MPa (as mentioned in the datasheet) and a Young’s modulus of 2630 ± 11.8 MPa compared to the 3D-printed samples. This can be attributed to porosity and interlayer adhesion in the printed samples. The low layer adhesion is due to the fact that the deposited printing ribbon cools down and then has to be partially re-melted by the overlying printing ribbon in order to create a bond between the printing layers. The highest deviation is related to the type of failure. The printed samples exhibit brittle failure, as expected for amorphous thermoplastic materials. The injection-molded samples show ductile failure with a high fracture strain, which is similar to the typical failure of semi-crystalline thermoplastic materials such as polyamide. Table 4 summarizes the used the achieved mechanical stiffness, fracture stress, and fracture strain related to injection- molded samples.

**Figure 12:**
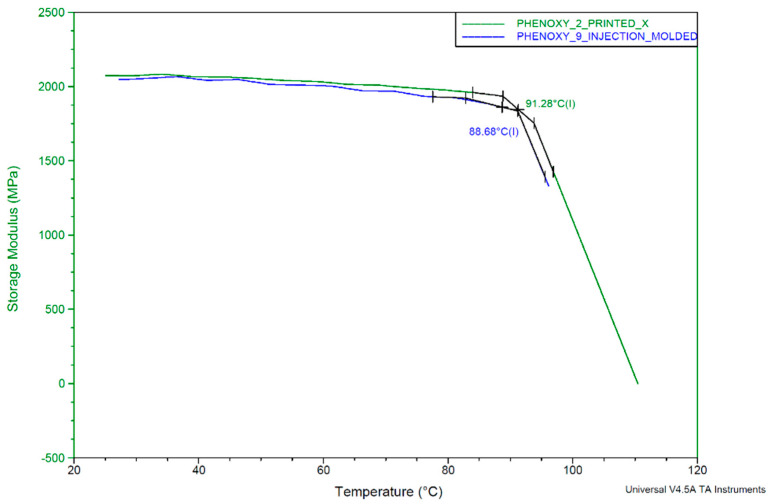
Result of dynamic mechanical analysis (DMA) measurement. Green curve: 3D printed, blue curve: injection molded.

To explore the temperature dependence behavior, mainly, the effect of the temperature on the stiffness, dynamic mechanical analysis (DMA) was performed with printed and injection-molded samples. A Q800 DMA measuring instrument from the manufacturer TA Instruments was used for the measurement. The dimensions of the sample were 32 mm length, 10 mm width, and 5 mm thickness. The measurement was performed at a frequency of 0.1 Hz and with a temperature ramp of 3 °C/min starting at 25 and rising to 115 °C. Figure 12 shows a comparison between a 3D-printed and an injection-molded specimen.

The result shows softening, due to the drop in storage modulus as measured by the onset, at about 91 °C for the printed test specimen and at about 89 °C for the injection-molded one. From room temperature up to the glass transition temperature, the stiffness is barely influenced by the temperature, which can be interpreted as a benefit of this material compared to others such as PA12, PLA, and ABS.

The variation of the glass transition shows a small difference between the 3D printed and the injection-molded specimen.

## 5. Discussion

The following Table 5 shows a comparison between phenoxy and the standard FFF materials polylactide acid (PLA), polyamide 12 (PA12), and acrylonitrile butadiene styrene (ABS). Of course, there are many different grades of PLA, ABS, and PA12, and the mentioned values are only a rough comparison of the performance to other materials; for detailed information, please review [6,11,12,13].

The glass transition temperature of phenoxy is above that of PLA and PA12 but below that of ABS. In addition, the material can be processed at a lower temperature. The filament price of the phenoxy here is an estimate based on the granule price. The material strength is higher than ABS and comparable to PA12 but lower than PLA [11,12,13].

In summary, the performed study shows an interesting material candidate for FFF with good processing capabilities such as reduced processing temperature and therefore a reduction of process induced warpage (50 °C difference in the processing temperature compare to ABS for instance) and good mechanical performance (nearly constant Youngs modulus up to 85 °C).

## 6. Conclusions

In this study, phenoxy was applied to FFF. It was characterized using DSC, TGA, DMA, and rheometric analysis. The glass transition temperature was around 95 °C, the material showed a shear thinning behavior, and degradation started above 300 °C.

A filament was produced according to the described process parameters with an average diameter of 1.74 mm variating by ± 0.09 mm. The extrusion was quite stable with an output rate of 1.6 kg/h, a pulling speed of 0.15 m/s, and an applied shear rate of 300 s^−1^.

This filament was used with a modified Ultimaker 2+ Extended FFF printer, and processing parameters such as the layer height (0.05 mm), layer width (0.4 mm), and nozzle temperature (200 °C) were used to print tensile bars according to DIN EN ISO standard 527-1. The phenoxy was printed with a maximum printing speed of 20 mm/s. The processing behavior was quite stable, and the resulting warpage was less related to the amorphous character of the polymer. Due to the lack of crystallization, there is less material shrinkage, which reduces warping.

Tensile tests in three different directions were performed to evaluate the mechanical characteristics, and these results were compared to the injection-molded material. Using FFF, the material does not have the same performance as the injection-molded material, but nevertheless, in the printing direction, the strength is high with 48.12 ± 3.12 MPa.

This study is only a first evaluation of the properties. A variation of process condition, other test methods such as the bending test, impact test, or other Phenoxy grades with different molecular weight distributions could also lead to an improvement of properties.

A potential interesting applications for this polymer can be elucidated by reviewing the applications in which the material is already used such as toughening of thermoset resin systems [14] or Phenoxy fibers as hot melt adhesive [15]. Examples include toughening applications because the material can dissolve into epoxide systems. A common standard is to dissolve phenoxy powder into brittle thermosets to increase fracture toughness [16]. Using this new possibility for additive manufacturing, other forms of gradient that applied toughening or hierarchical structures could be of interest for further research.

A further possibility of application is related to a study of Zweifel et al. During the curing of a thermoset composite, a phenoxy layer was co-cured as a boundary layer and used for resistance welding [17]. Given the design freedom of additive manufacturing and this new joining technology, an enabling method could be applied.

## Figures and Tables

**Table 1:** Processing parameters for the filament production.

Extruder zone 1 (Feed)	30 °C
Extruder zone 2 (Rear)	195 °C
Extruder zone 3 (Center)	195 °C
Extruder zone 4 (Front)	195 °C
Extruder zone 5 (nozzle)	195 °C
Water reservoir 1	30 °C
Water reservoir 2	20 °C
Extruder speed	35 U/min
Pressure	42 bar

**Table 2:** Processing parameters for tensile bars in the FFF process.

Layer height	0.05 mm
Layer width	0.4 mm
Infill	100 %
Nozzle temperature	200 °C
Printing speed	20 mm/s
Bed temperature	60 °C

**Table 3:** Average result with standard deviation of tensile test of 3D printed samples.

Printing Direction	Fracture Stress σ_b_	Fracture Strain ε_b_	Young’s Modulus
X-direction	48.12 ± 3.17 MPa	2.15 ± 0.27%	2594.14 ± 77.34 MPa
Y-direction	40.80 ± 6.00 MPa	1.79 ± 0.32%	2495.68 ± 87.91 MPa
Z-direction	8.89 ± 0.74 MPa	0.39 ± 0.05%	2203.14 ± 136.71 MPa

**Table 4:** Average result with standard deviation of tensile test of injection-molded samples.

Yield Stress σ_y_	Yield Strain ε_y_	Fracture Stress σ_B_	Fracture Strain ε_y_	Young’s Modulus E
65.53 ± 0.76 MPa	5.26 ± 0.05%	45.83 ± 0.96 MPa	38.57 ± 5.60%	2634.03 ± 11.79 MPa

**Table 5:** Comparison between phenoxy and standard FFF materials.

	Phenoxy	PLA (2015, [6,13])	PA12 (2022, [6,13])	ABS (2019, [6,13])
Glass transition temperature (°C)	96.65 ± 0.93 °C	60	49	105
Melting temperature (°C)	-	170	175	-
Processing temperature (°C)	200	210	235	250
Heat bed temperature (°C)	60	60	120	60
Approx. filament price (Fr/kg)	25 *	35	72	73
Strength in printing direction σ_x_ (MPa)	48.12 ± 6.00	58–85	35–50	32–40
Fracture strain in printing direction ε_Bx_ (%)	2.15 ± 0.27	4	5	2
Young’s modulus in printing direction E_x_ (MPa)	2594.14 ± 77.34	2780–4300	1400	1600–1960

* Approximation.

## Data Availability

The data presented in this study are available on request from the corresponding author.
